# The Husavirus Posa-Like Viruses in China, and a New Group of *Picornavirales*

**DOI:** 10.3390/v12090995

**Published:** 2020-09-07

**Authors:** Zhenzhi Han, Jinbo Xiao, Yang Song, Mei Hong, Guolong Dai, Huanhuan Lu, Man Zhang, Yueling Liang, Dongmei Yan, Shuangli Zhu, Wenbo Xu, Yong Zhang

**Affiliations:** 1 WHO WPRO Regional Polio Reference Laboratory and NHC Key Laboratory for Biosafety, NHC Key Laboratory for Medical Virology, National Institute for Viral Disease Control and Prevention, Chinese Center for Disease Control and Prevention, Beijing 102206, China; hansir8@ivdc.chinacdc.cn (Z.H.); mr_mint1114@ivdc.chinacdc.cn (J.X.); candyalbarn57@ivdc.chinacdc.cn (Y.S.); luhuanhuan0908@ivdc.chinacdc.cn (H.L.); 952135006@ivdc.chinacdc.cn (M.Z.); liangyueling1994@ivdc.chinacdc.cn (Y.L.); dongmeiyan1976@ivdc.chinacdc.cn (D.Y.); zhusl@ivdc.chinacdc.cn (S.Z.); wenbo_xu1@ivdc.chinacdc.cn (W.X.); 2 Tibet Center for Disease Control and Prevention, Tibet Autonomous Region, Lhasa 850000, China; 13989900080@139.com (M.H.); 18387420919@163.com (G.D.); 3 Center for Biosafety Mega-Science, Chinese Academy of Sciences, Wuhan 430071, China

**Keywords:** husavirus, posa-like virus, Picornavirales, phylogeny, evolution

## Abstract

Novel posa-like viral genomes were first identified in swine fecal samples using metagenomics and were designated as unclassified viruses in the order *Picornavirales*. In the present study, nine husavirus strains were identified in China. Their genomes share 94.1–99.9% similarity, and alignment of these nine husavirus strains identified 697 nucleotide polymorphism sites across their full-length genomes. These nine strains were directly clustered with the Husavirus 1 lineage, and their genomic arrangement showed similar characteristics. These posa-like viruses have undergone a complex evolutionary process, and have a wide geographic distribution, complex host spectrum, deep phylogenetic divergence, and diverse genomic organizations. The clade of posa-like viruses forms a single group, which is evolutionarily distinct from other known families and could represent a distinct family within the *Picornavirales*. The genomic arrangement of *Picornavirales* and the new posa-like viruses are different, whereas the posa-like viruses have genomic modules similar to the families Dicistroviridae and Marnaviridae. The present study provides valuable genetic evidence of husaviruses in China, and clarifies the phylogenetic dynamics and the evolutionary characteristics of *Picornavirales*.

## 1. Introduction

The order *Picornavirales* is composed of eight families (*Picornaviridae*, *Dicistroviridae*, *Marnaviridae*, *Iflaviridae*, *Polycipiviridae*, *Caliciviridae*, *Solinviviridae*, and *Secoviridae*) as well as other unclassified picornaviruses [1]. *Picornavirales* pathogens are associated with a wide range of infectious diseases, including hepatitis and hand, foot, and mouth disease (HFMD) in humans, foot-and-mouth disease (FMD) in animals (pigs, goats, cattle, and other animals), and plant diseases (e.g., Tomato torrado disease and Satsuma dwarf disease) [2]. These pathogenic *Picornavirales* usually infect a broad range of hosts, including arthropods, insects, algae, humans, monkeys, and other organisms [3]. *Picornavirales* have a positive sense ssRNA genome between 7.2 and 10.2 kb, which encodes a polyprotein cleaved by proteases; however, some plant-infecting picornaviruses (Secoviridae) possess segmented RNA genomes. The genomic nucleotide sequences of *Picornavirales* are highly divergent, and their genomic organization models are highly variable among different families. The polyprotein of *Picornavirales* usually contains a conserved replication block of helicase, protease, and RNA-dependent RNA polymerase (Hel-Pro-Pol) [2]. There are three typical genomic organizations observed in the order *Picornavirales*. The first genomic organization has the non-structural module (NS-module) located at the 5′ end of the genomic sequence and the structural module (S-module) located at the 3′ end, separated by an intergenic region (as in the families Dicistroviridae and Marnaviridae). Similar genomic organization is observed in the family Secoviridae, except that the two modules are located on different genomic segments. In the third genomic organization, the S-module is at the 5′ end of the genome, whereas the NS-module is at the 3′ end, as observed in Picornaviridae, Iflaviridae, and Polycipiviridae [2,3].

With the development of deep transcriptome sequencing, more novel unclassified RNA virus genomes have been identified, redefining the proposed evolutionary progress of the virosphere [4,5]. Following breakthrough research, more potential viromes or viral pathogens have been identified and expanded upon [6,7,8]. The unprecedented diversity and evolutionary scale of viromes have been analyzed and illustrated, offering deep insights into their evolutionary history [9]. The pathogens in *Picornavirales* have been recently expanded, with more divergent genomes identified and analyzed [4,5,10,11,12,13,14]. The relationship between the *Picornavirales* and their diseases is unclear, except for the culturable and disease-causing agents. The viral genomic organization patterns, host ranges, and geographic distribution of *Picornavirales* are diverse and contribute to their pathogenicity.

Novel posa-like virus genomes were first identified in swine fecal samples using metagenomics and were assigned as unclassified viruses to the order *Picornavirales* [12]. Further reports of novel posaviruses with low amino acid sequence identity revealed novel genomic organization features and phylogenetic characteristics of posa-like viruses [15,16]. In China, posaviruses were detected in fecal samples from pigs with diarrheal signs caused by unspecified pathogens [17,18]. The posa-like viruses have been detected in specimens from a broad range of hosts, such as the fish stool-associated RNA virus (fisavirus), human stool-associated RNA virus (husavirus), panda stool-associated RNA virus (pansavirus), bat stool-associated RNA virus (basavirus), and rat stool-associated RNA virus (rasavirus) [11,16,19]. Due to high sequence similarity between posavirus strains and parasite-derived genomic sequences, it was speculated that posaviruses could not infect swine, but instead may have a dietary or environmental origin [12]. However, the host of husavirus remained unclear, even though the virus was detected in human stool samples [11]. Most posa-like viruses were identified in the stool samples of animals, whereas limited surveys of husavirus have been reported worldwide [11,16,20]. To the best of our knowledge, there are no reports of husavirus in China to date, and their genetic and phylogenetic characteristics remain unknown.

In the present study, we first identified nine husavirus strains in China with high genomic similarity. The genomic characteristics, phylogenetic relationships, and genomic arrangements of these viruses revealed the detailed evolutionary lineage of husavirus. We also investigated the diversity of posa-like viruses and showed they form a separate clade within the *Picornavirales.* Outcomes of this study provide a valuable genetic evidence about husavirus in China and comprehensive information on the evolutionary characteristics of *Picornavirales*.

## 2. Materials and Methods

### 2.1. Ethics Statement and Sample Collection

Human stool samples were collected from healthy children. In total, 91 fecal samples were obtained during public health surveillance. Written informed consent for the analysis of their clinical samples was obtained from the parents or guardians of the children included in the present study. This study was approved by the Ethics Review Committee (IVDC2016-004, February 2016) of the National Institute for Viral Disease Control and Prevention (IVDC), Chinese Center for Disease Control and Prevention. All experimental protocols were approved by the IVDC, and the methods were carried out in accordance with the approved guidelines [21].

### 2.2. Library Preparation and Metagenomic Sequencing

Fecal samples were processed using a previously published method [22,23]. Total RNA was extracted from enriched virus-like particles using a QIAamp Viral RNA Mini Kit (Qiagen, Hilden, Germany). The extracted RNA of all samples was pooled for library construction, followed by amplification using the REPLI-g Cell WGA & WTA Kit (150052; Qiagen, Hilden, Germany). Amplified DNA was randomly fragmented by ultrasound sonication (Covaris M220, Woburn, MA, USA) to produce 800 bp fragments, then sticky ends were repaired and adapters were added using T4 DNA polymerase (M4211, Promega, Madison, WI, USA), Klenow DNA Polymerase (KP810250, Epicentre, Woburn, MA, USA), and T4 polynucleotide kinase (EK0031, Thermo Scientific, Fermentas, GlenBurnie, MD, USA). Each viral sequencing library was prepared following the Illumina TruSeq DNA Preparation Protocol and was sequenced on the HiSeq4000 platform (Illumina, San Diego, CA, USA), with 150 bp paired ends. The library preparation and sequencing process was performed by BGI Tech (Shenzhen, China).

### 2.3. Quality Control, Assembly, and Analysis

Low-quality bases (PHREAD q < 20) and adaptors were trimmed using Trimmomatic software (version 0.39) [24]. Clean reads were aligned to the human reference genome (hg19), and reads matching the human genome were discarded [25]. The remaining reads were de novo assembled using Trinity software (version 2.5.1), and taxonomically assigned using Centrifuge (version 1.0.4) for metagenomic classification [26,27]. The assembled contigs were taxonomically assigned using the BLASTn algorithm (https://blast.ncbi.nlm.nih.gov/Blast.cgi), with an e-value cut-off of 1 × 10^−5^. We identified the viral annotation of posa-like viruses by manually inspecting the BLAST results and the taxonomic results from Centrifuge. To confirm the assembled contigs, clean reads were mapped to the reference genome of husavirus (GenBank accession number KX673274.1) using Bowtie2 (version 2.3.4.3) [25]. Finally, we manually checked the mapped results and compared them with the assembled contigs.

### 2.4. Detection and Molecular Typing of Novel Husaviruses

The assembled library was used to identify husaviruses by real-time (RT)-PCR assays using previously described husavirus-specific probes and primers [11]. After confirming husavirus in a sample, RT-PCR was performed to amplify the partial coding region using the PrimeScript One Step RT-PCR Kit Ver.2 (TaKaRa, Dalian, China) with specific primers (Table S1, Supplementary Materials). The PCR products were purified using a QIAquick PCR purification kit (Qiagen, Hilden, Germany). The ABI 3130 Genetic Analyzer (Applied Biosystems, Foster City, CA, USA) was used for sequencing in both directions. The acquired partial genomic sequences were analyzed using BLAST against the GenBank database. A total of nine husavirus strains were confirmed based on their sequence information.

### 2.5. Full-Length Genome Sequencing of Nine Husavirus Strains

The full-length genome sequences of nine husavirus strains were amplified using the “primer-walking” strategy, which was used to close the gaps in the sequence. Briefly, the overlapping fragments representing whole genomes were amplified by RT-PCR using specific primers (Table S1). The RT-PCR products were purified for sequencing using the QIAquick Gel extraction kit (Qiagen, Hilden, Germany) and the amplicons were sequenced on the ABI 3130 Genetic Analyzer (Applied Biosystems, Foster City, CA, USA) as described above. The 3′ end of the genome was amplified using an oligo-dT primer as reported previously [28]. The 5′ end of the genome was amplified using the 5′-Full RACE Kit (Takara, Shiga, Japan) and following the manufacturer’s instructions. Sequencher software (version 5.0, Ann Arbor, MI, USA) was used to assemble the contigs with the reference genome and to produce the consensus sequences.

### 2.6. Genome Annotation Characteristics and Phylogenetic Analysis

**Figure 1:**
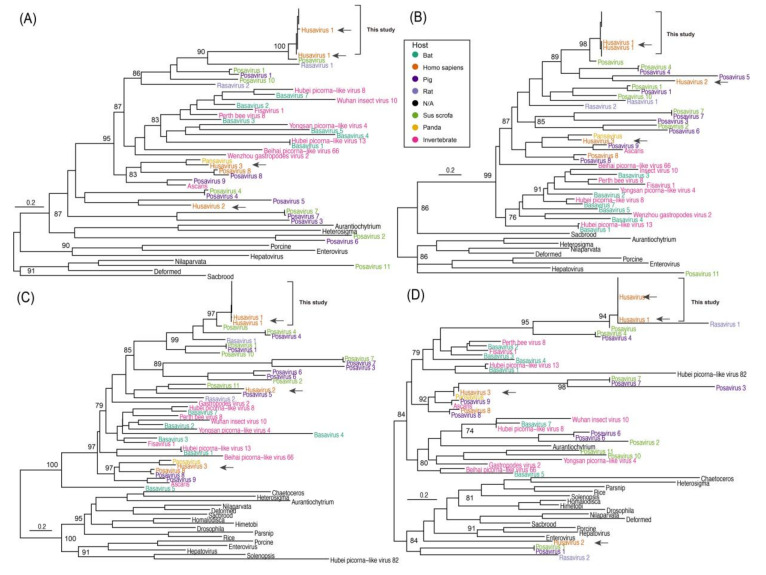
Maximum-likelihood phylogenetic tree of different conserved domain amino acid sequences. (**A**–**D**) show the phylogenetic trees based on the picorna-like capsid protein domain 1, picorna-like capsid protein domain 2, RNA-dependent RNA polymerase, and helicase, respectively. The black arrows represent the major husaviruses reported, and the box shows the husavirus strains besides the previously reported Husavirus 1. The genomes of six known families of *Picornavirales* were used as outgroups. The names are colored according to the types of hosts in the inset. Scale bars indicate the substitutions per site per year. The numbers at each node indicate the bootstrap support value, with 1000 bootstrap replicates.

The open reading frame (ORF) was determined using ORFfinder software (https://www.ncbi.nlm.nih.gov/orffinder/?tdsourcetag=s_pctim_aiomsg) for nearly full-length genomic sequences of the nine husavirus strains. Combined with previous reports on the genomic organization of husaviruses, we identified the ORF length and deduced the amino acid sequences. To infer the possible protein-coding domain of the novel genome, an RPS-BLAST search against the conserved domain database (CDD) was performed [29]. The husavirus RNA-dependent RNA polymerase (RdRp), helicase, 3C cysteine protease, and picorna-like capsid protein domains were identified. For other posa-like viral genomes, we applied a similar strategy to obtain the sequence information for major protein domains, even though some annotations of posa-like viruses failed due to the vast genomic divergence among these viruses. Representative posa-like virus strains were selected based on the phylogenetic relationships of the conserved domain sequences and previous reports [16,30]. The respective protein sequences of different functional domains were extracted and incorporated into the subsequent analysis. Based on the phylogenetic relationships within the RdRp domain, we extracted and analyzed the full-length genomes that were similar to Husavirus 1 (Figure 1C). The full-length genomic sequences of Husavirus 1–3 and the neighboring posa-like viruses were used to construct the maximum-likelihood phylogenetic tree.

The obtained amino acid sequences were aligned using MAFFT software (version 7.407), with the E-INS-I algorithm [31]. The remaining ambiguously aligned regions were removed using the TrimAl program [32]. The maximum-likelihood phylogenetic tree was constructed using IQ-TREE software (version 1.6.12), with 1000 bootstrap replicates, and the best amino acid substitution models were inferred with ModelFinder, using Bayesian information criteria [33,34,35]. We manipulated the phylogenetic tree topology for clear display using the ggtree package [36]. Although the actual hosts of many posa-like viruses remain unknown, the hosts where they were initially identified and their regional information were included in the discriminant analysis (DA). The RdRp protein sequences were used to infer the geographic and host clustering, which was implemented in the discriminant analysis of the principal component analysis (PCA) using the adegenet package [37,38].

### 2.7. Data Availability

The full-length genomic sequences for the nine strains identified in the present study were deposited in the GenBank nucleotide sequence database under accession numbers MT586615–MT586623, and the metagenomic data were submitted to the NCBI’s Sequence Read Archive (SRA) under accession number SRP266688.

## 3. Results

### 3.1. Discovery of Husavirus in China

**Figure 2:**
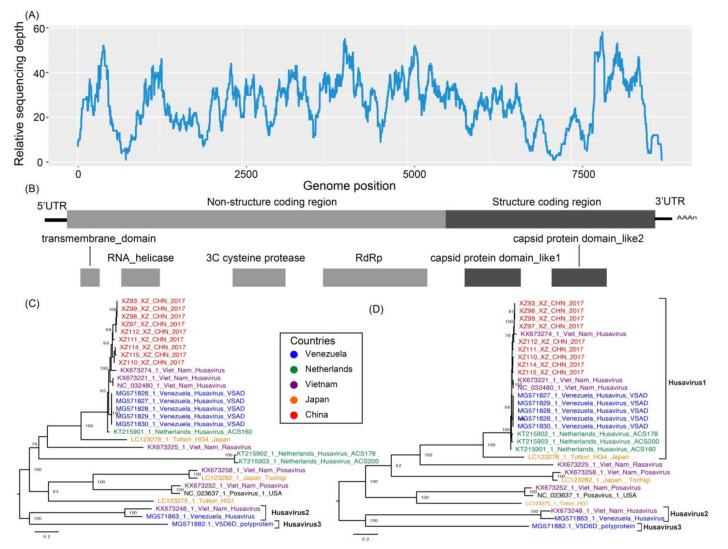
(**A**) Relative sequencing depth across the reference genome. (**B**) The classical genomic organization of posa-like viruses. (**C**) The maximum-likelihood phylogenetic tree of husaviruses and the neighboring posa-like viruses, constructed using nucleotide sequences. (**D**) The maximum-likelihood phylogenetic tree of husaviruses and the neighboring posa-like viruses was constructed using amino acid sequences. Scale bars indicate the substitutions per site per year, and taxa are colored according to the countries of origin. The box show the previously reported Husavirus 1 strain. The numbers at each node indicate the bootstrap support value, with 1000 bootstrap replicates.

After trimming the raw reads, 32,780,844 clean reads with a Q20 larger than 98% were obtained. By mapping to the host genome, about 60% of the clean reads were removed and 13,112,337 clean reads were used for de novo assembly. Finally, 29,868 assembled contigs were obtained, 303 of which were larger than 3 kb. We compared the assembled contigs against the nucleotide database with a threshold E-value of 1 × 10^−5^, resulting in 284 assembled contigs under viral annotation. Finally, we identified two nearly full-length genome sequences for husavirus, which belong to the order *Picornavirales*, sharing 93% genomic identity with strain 19344_29 (GenBank accession number KX673274). To confirm the identity of these assembled contigs, we mapped the clean reads back to the genome of husavirus (GenBank accession number KX673274). In total, 1350 clean reads were mapped to the reference genome when the repetitive reads were excluded, and the mean sequencing depth was 22 (Figure 2A).

### 3.2. Full-Length Genomic Characterization of Nine Husavirus Strains

We used real-time (RT)-PCR assays to detect husaviruses in all samples used to construct the library, using a previously published probe and primer [11]. Nine clinical samples were positive for husavirus, with cycle threshold (CT) values ranging from 18 to 31. All patients whose clinical samples were positive were less than five years old. These children included two boys and seven girls from different counties within the same prefecture.

Full-length genome sequences of the nine husavirus strains were determined using Sanger sequencing and a “primer-walking” strategy. All strains were 9003–9009 nt in length, with a poly(A) tail. Alignment of the nine husavirus strains identified 697 nucleotide polymorphic sites across the full-length genome. Strains XZ114_XZ_CHN_2017 and XZ115_XZ_CHN_2017 contained a six-nucleotide deletion at position 8221, resulting in a two amino-acid deletion at position 2722 of the protein sequence. The ORF of the nine strains was 8813–8819 nt in length, encoding a polypeptide of 2970–2972 amino acids, with a 5′-UTR of 53 nt and 3′-UTR of 37 nt. The overall base composition of the nine strains was 21.8–22%A, 23.8–24%C, 29–29.4%G, and 24.9–25%T. The full-length genome nucleotide and amino acid similarity among the nine strains was 94.1–99.9% and 96.8–100%, respectively (Table S2, Supplementary Materials).

To assess the divergence between the nine strains, nucleotide variation was analyzed using the strain 19344_29 isolated from Vietnam (GenBank accession number KX673274) as a reference strain (Figures S1 and S2, Supplementary Materials). The genome sequence of each strain had 93.2–93.8% nucleotide identity and 96.5–97.5% amino acid identity with the strain 19344_29. The nine husavirus strains diverged at 600, 3100, and 7000–9000 nt across the entire genome compared with the strain 19344_29, indicating that evolution occurred during their circulation, despite their relatively close geographical distribution (Figures S1 and S2). We observed slight differences in the nucleotide sequences of the husavirus strains, implying that nucleotide substitution had occurred, although the strains were sometimes found in the same prefecture.

### 3.3. Phylogenetic Comparison of Husavirus with Other Posa-Like Genomes

Due to the low genomic sequence similarity between the husavirus and unclassified posa-like viruses in *Picornavirales*, it was difficult to perform a phylogenetic analysis at the full-length genome level. Therefore, we identified the conserved domains of the protein sequences, which included the RNA-dependent RNA polymerase (RdRp), helicase, 3C cysteine protease, and picorna-like capsid protein domains. Since some posa-like viral protein sequences could not be annotated as the major domains in the CDD, the genomes with invalid annotations were discarded. We obtained the representative lineages of the posa-like viruses from previous publications [16,30]. Based on the conserved protein sequences of posa-like viruses, maximum-likelihood phylogenetic trees were constructed to explain the phylogeny of husaviruses (Figure 1). The posa-like viruses showed high diversity and the topology of the phylogenetic tree was variable when we used the six known families of *Picornavirales* as the outgroup. The posa-like viruses formed a single group in the phylogenetic tree and presented complex varieties based on different domains.

The nine husavirus strains in the present study clustered with Husavirus 1 (GenBank accession numbers KT215902 and KX673221), and showed close phylogenetic association with Posavirus (GenBank accession number LC123278) in all the maximum-likelihood trees except for the tree based on the helicase domain, indicating that the husavirus strains in the present study belong to the Husavirus 1 lineage (Figure 1). The Husavirus 1–3 lineages did not cluster together in each phylogenetic tree, indicating significant divergence and an intricate evolutionary history among the husaviruses (Figure 1, black arrows). The Husavirus 1–3 lineages diverged a long time ago, even though they were identified recently in human fecal samples. The Husavirus 1 lineage is widespread globally, because the distant strains are closely clustered together. For example, the strains identified in Vietnam, Netherlands, China, and Venezuela clustered together (Figure 2C,D). We observed that the strains from China and Vietnam were very similar, revealing that husavirus possibly co-circulated in Tibet via China and Vietnam.

Although several posa-like viruses were identified in the stool samples of pigs, their hosts were varied and complex, ranging from invertebrates to vertebrates (Figure 1). Surprisingly, the strain HG4 (GenBank accession number LC123278) identified in *Sus scrofa* and the strain 16715_36 identified from rats showed a close phylogenetic relationship with the known Husavirus 1 lineage (Figure 2C,D). This was also observed in other posa-like viruses (e.g., Fisavirus 1 and Basavirus 3). Collectively, our results show that these viruses have close phylogenetic relationships but they also have a varied and wide host spectrum.

### 3.4. Genomic Organization of the Husaviruses

**Figure 3:**
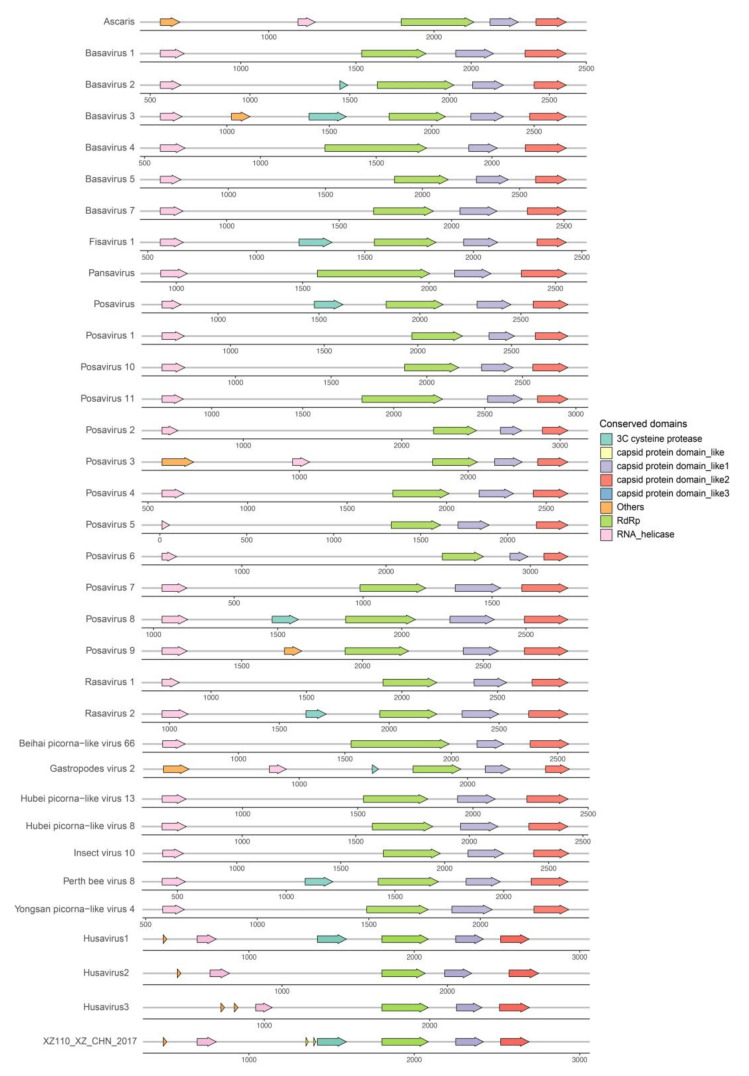
The genomic organization of the representative posa-like viruses and a representative husavirus identified in the present study. The arrow blocks show the conserved protein domains and their positions along the genome. Different colors indicate different protein domains. All conserved domains are drawn to scale with the genome size. For clear display, the label “others” refers to the non-conserved domains, including the Picornavirus core protein 2A, Peptidase_C3G superfamily, Poliovirus 3A protein like domain, and CRPV capsid protein-like domains.

Several major conserved domains of the Husavirus 1–3 lineages are located at different genomic positions, indicating different evolutionary directions and intricate evolutionary history. The husavirus strains identified in the present study have the same genomic organization as the Husavirus 1 lineage, confirming the phylogenetic results based on the major conserved domains. The replication block of the helicase, protease, and RNA-dependent RNA polymerase (Hel-Pro-Pol) was identified in every husavirus genome obtained, which is consistent with the classical conservative modules of *Picornavirales*. We compared the representative genomic arrangements of different posa-like viruses, and found that the replication block of Hel-Pro-Pol exists in all posa-like viruses except in the partial failed annotations of the protease domains (Figure 3). Two capsid protein domains were also identified in each of the representative posa-like viral genomes, which verified the common genetic characteristics of the genomic arrangements of posa-like viruses. The posa-like viral genomes had a non-structural module (NS-module) at the 5′ end and a structural module (S-module) at the 3′ end, which were similar between strains, with some deviation in the coding region (Figure 2B and Figure 3). Changes in the location of the main functional domains in posa-like viruses were observed, suggesting genomic rearrangements have occurred.

### 3.5. Identification of a New Group of *Picornavirales*

**Figure 4:**
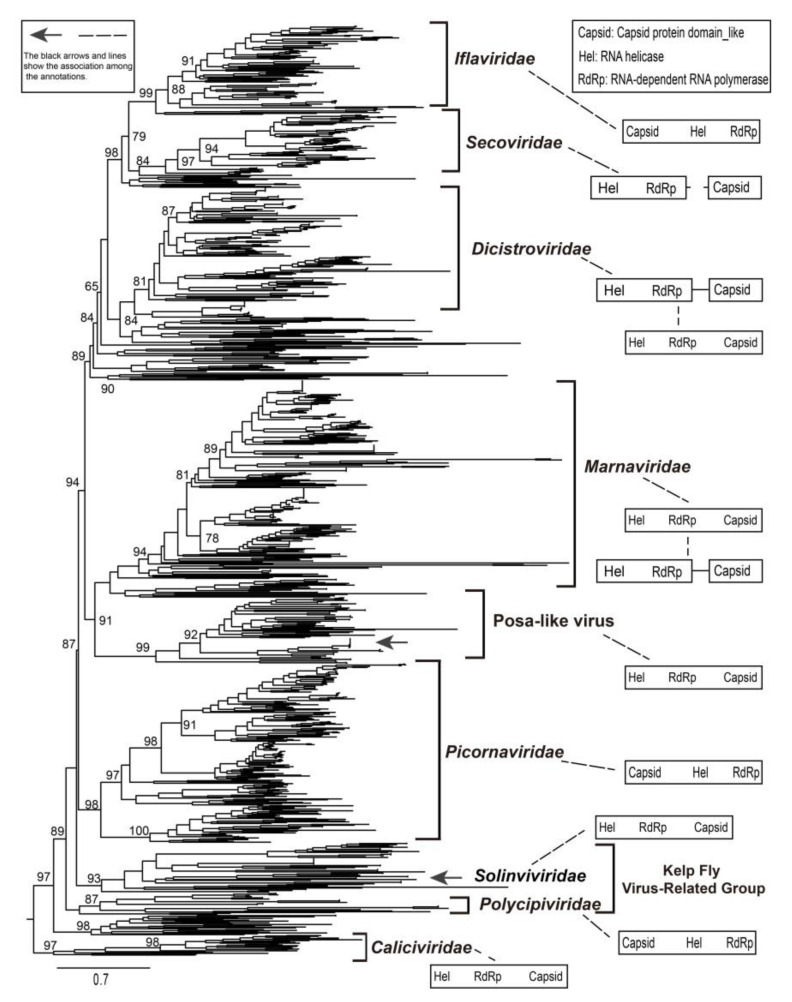
The maximum-likelihood phylogenetic tree of posa-like viruses, other known families, and unclassified viruses of *Picornavirales*. The conserved amino acid sequences of RdRp were used to generate phylogenetic trees. Scale bars indicate the substitutions per site per year. The numbers at each node indicate the SH-like approximate likelihood ratio test (SH-aLRT) support value, with 1000 iterations. The black arrows represent the husavirus strains identified in the present study. The genomic organization on the right shows the major non-structural and structural domains, with the arrangement of open reading frames (ORFs) based on previous reports [2,3,5].

We used the representative conserved RdRp sequences of *Picornavirales* obtained from the GenBank to assess the evolutionary history of *Picornavirales* [2,3,4,5]. The *Picornavirales* sequences presented extremely divergent characteristics suggesting a long evolutionary time scale. Posa-like viruses identified in previous reports and in the present study formed a single group and clustered with the genomes of family Marnaviridae (Figure 4). Furthermore, the posa-like viruses had distant phylogenetic associations with other families belonging to the order *Picornavirales*. The families Iflaviridae, Secoviridae, Dicistroviridae, and novel branches clustered together to form a large clade. A novel group (e.g., kelp fly virus-related group), which contained the genomes of the known families Polycipiviridae and Solinviviridae, was identified [5,11]. The presence of clades outside those of the defined families of *Picornavirales* allowed the identification of novel groups and the definition of their phylogenetic relationships. For instance, the unknown clades located between the Dicistroviridae and Marnaviridae imply that novel *Picornavirales* may have existed, or may still exist (Figure 4).

As several novel genomes of *Picornavirales* have been found, the arrangement of ORFs and the order of non-structural and structural genes were investigated (Figure 4). The genomic organization of Picornaviridae, Iflaviridae, and Polycipiviridae was similar, whereas the families Dicistroviridae and Marnaviridae shared the same genomic module models, with the NS-module located in the 5′ end of their genomes. The genomes of family Secoviridae were separated into two segments. The genomic arrangement of the posa-like viruses was similar to that of families Marnaviridae and *Dicistroviridae*, in which the former was frequently identified from marine phytoplankton (e.g., algae). The phylogeny of posa-like viruses confirmed their close relationship with the family Marnaviridae, thereby providing valuable information about the origin of posa-like viruses.

### 3.6. Host and Geographic Clustering Characteristics

**Figure 5:**
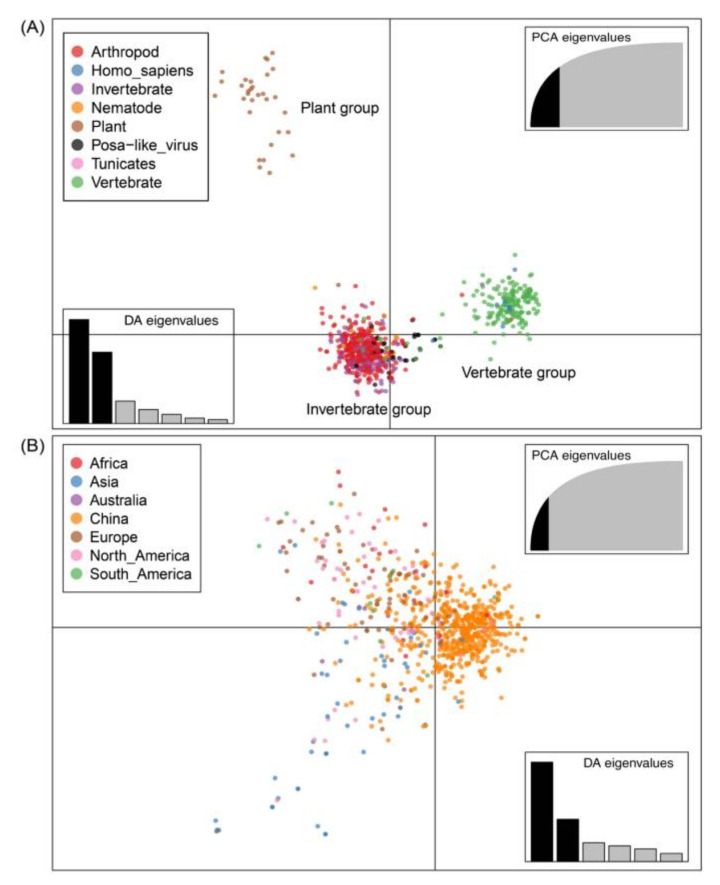
The first two principal components of the genomic sequences of *Picornavirales*, with the hosts and available sample locations used as the prior groups. Eigenvalues of the analysis (PCA and DA) are displayed in the inset, along which the black modules represent the dimensions retained and the gray modules show the dimensions eliminated in the datasets. Groups are shown in different colors, with dots representing individual strains. (**A**) The scatterplot using the host information as a prior cluster. (**B**) The scatterplot using the location information as a prior cluster. PCA, principal component analysis; DA, discriminant analysis.

Significant separation was observed across three major clusters (plant, invertebrate, and vertebrate groups), with the host information used as prior clusters (Figure 5A). The strains from plants formed a single cluster, whereas strains from vertebrates and humans formed one cluster. The viruses from other hosts including arthropods, invertebrates, nematodes, and tunicates were clustered together, with the arthropods dominating. The groups of viruses identified in invertebrate and vertebrate hosts have close evolutionary relationships, with partial mixing. The posa-like viruses cluster within the invertebrate host group, suggesting a possible common origin. However, we did not observe a distinct divergence when location information was used as the prior cluster (Figure 5B). This indicates that the individual strains from different regions are more similar than strains found in different hosts, confirming significant overlap between different regions.

## 4. Discussion

With the development of next-generation sequencing and its application in pathogen detection, the virosphere is being explored beyond the limits of culturable pathogens [9]. The number of genomes belonging to the order *Picornavirales* has sharply increased as many divergent genomic sequences and undiscovered viromes have been identified [4,5]. Several members of the order *Picornavirales* are pathogenic, and can cause devastating economic consequences [1,2,3]. The host spectrum of the order *Picornavirales* is wider than expected, and includes plants, algae, insects, and vertebrates. Although the replication block of Hel-Pro-Pol is conserved in *Picornavirales*, the genomic arrangement of *Picornavirales* seems to be extremely flexible [3]. The order of the NS- and S-modules, as well as the arrangement of ORFs, are considerably variable across different families of *Picornavirales*.

The posa-like virus isolated from pig fecal samples in 2011 was previously unclassified in the order *Picornavirales* [12]. Although some studies had identified posa-like viruses in the stool samples of animals, information on the husaviruses remained limited [11,15,16,39], with no studies reported on husaviruses in China. In the present study, we identified husavirus strains in China by RNA sequencing. We found nine clinical samples positive for husavirus, and the full-length genomes were acquired from these stool samples. With different husavirus genomes identified simultaneously, our results confirm that husavirus is circulating in China.

The high nucleotide and amino acid sequence similarity among these nine strains showed that they were closely associated. Strains XZ114_XZ_CHN_2017 and XZ115_XZ_CHN_2017 included two amino acid deletions at the 3′ end of the coding region, and dominant divergence of the full-length genome at position 7000–9000 nt within the structural coding region, compared with strain 19344_29. A similar result was also observed upon comparing the husavirus strains identified in the present study with Husavirus 1 lineage strains. The co-circulation of Husavirus 1 lineage in China and Vietnam was confirmed, with some nucleotide substitutions identified between geographically close circulating strains.

Posa-like viruses appear to have gone through a long evolutionary progress, based on the complexity of their phylogenetic relationships. The nine strains identified in the present study clustered with the Husavirus 1 lineage, and their genomic organization was also similar to that of the Husavirus 1 lineage. Although the Husavirus 1–3 lineages were first found in human stool samples, they showed significant differences in their phylogenetic and genomic organization, indicating that they have diverged to some degree. The geographic distribution of husaviruses is wide, involving several distant countries; furthermore, husavirus-like viruses were identified in a broad spectrum of animals, ranging from invertebrates to vertebrates. Surprisingly, the strain HG4 (GenBank accession number LC123278) from *Sus scrofa* shared a closer phylogenetic relation with the Husavirus 1 lineage than other lineages. The conserved replication block of Hel-Pro-Pol existed in almost all posa-like viruses sequenced in the present study, and the two capsid protein domains were also identified in all posa-like viruses, indicating a common conserved genomic arrangement. We also identified different genomic organization features of posa-like viruses, particularly in the coding region.

The available posa-like virus sequences cluster near the family Marnaviridae. Our results confirmed the formation of a novel group within the *Picornavirales*, and posa-like viruses could be a distinct novel family within the *Picornavirales* [11,39]. A large number of novel branches, such as the kelp fly virus-related group, contain unclassified *Picornavirales* genomes. The genomic organization of *Picornavirales* is diverse, including different arrangement of ORFs as well as different orders of non-structural and structural coding regions. There was no apparent association between genomic organization and host or geographical location. The kelp fly virus-related group possessed the most diverse genomic arrangements, whereas the genomes of family Secoviridae were generally divided into two segments.

Based on discriminant analysis, we did not observe significant geographic clustering of viral lineages, whereas host-specific genomic clusters were evident. Three groups of hosts were identified for which the *Picornavirales* genomes had close evolutionary association. The posa-like viruses may have originally had an invertebrate vector although modern posa-like viruses have been identified in the stool of pigs, bats, fishes, pandas, and humans [11,16]. The lack of geographic clustering of *Picornavirales* suggests a wide distribution and complicated diffusion. Previous reports have shown that posavirus likely originated in an aquatic host, whereas fisavirus and basavirus possibly jumped to humans due to dietary or environmental contamination [12,15,16]. The picornavirus sequences identified in porcine stool samples shared high identity with the cDNA sequences derived from nematodes [12]. If we infer the evolutionary relationship of posa-like viruses through genomic organization and phylogeny, posa-like viruses appear to be closely associated with the family Marnaviridae. Our results suggest that undigested food, which could contain invertebrates or gut parasites, might be the source of posa-like viruses, which is consistent with previous studies.

## 5. Conclusions

To the best of our knowledge, this is the first study to report nine full-length genomic sequences of husaviruses identified for the first time in China. These husavirus strains provide the baseline data of their full-length genome features and phylogenetic characteristics. The genomic organization, entire genome features, and phylogenetic association with posa-like viruses were analyzed in detail, illuminating the dynamics of posa-like viruses. We explored the phylogenetic relationships of *Picornavirales* and speculated the possible origins of posa-like viruses. Overall, we provide comprehensive phylogenetic information to improve our understanding of the evolutionary history of *Picornavirales.*

## Supplementary Materials

The following are available online at https://www.mdpi.com/1999-4915/12/9/995/s1. Figure S1: Sequence similarities analysis of husavirus strains with the reference strain (KX673274.1_Husavirus_isolate_19344_29). Figure S2: Nucleotide variation across the genome of nine husavirus strains. Table S1: The primers used for amplification and sequencing. Table S2. The genomic sequence identity percentage of nucleotide and amino acid sequences, including the nine strains in this study and a reference strain 19344_29 (GenBank accession number KX673274).
