# Supplementary material for: The Husavirus Posa-Like Viruses in China, and a New Group of Picornavirales
Source: Viruses. 2020 Sep 7;12(9):995. doi: 10.3390/v12090995 (PMC7551994; doi:10.3390/v12090995)
Supplement: Supplementary file 1 [file viruses-12-00995-s001.zip › viruses-877899-supplementary.pdf]

Figure S1. Sequence similarities analysis of husavirus strains with the reference strain (KX673274.1_Husavirus_isolate_19344_29). The strain 19344_29 was used as the query sequence.

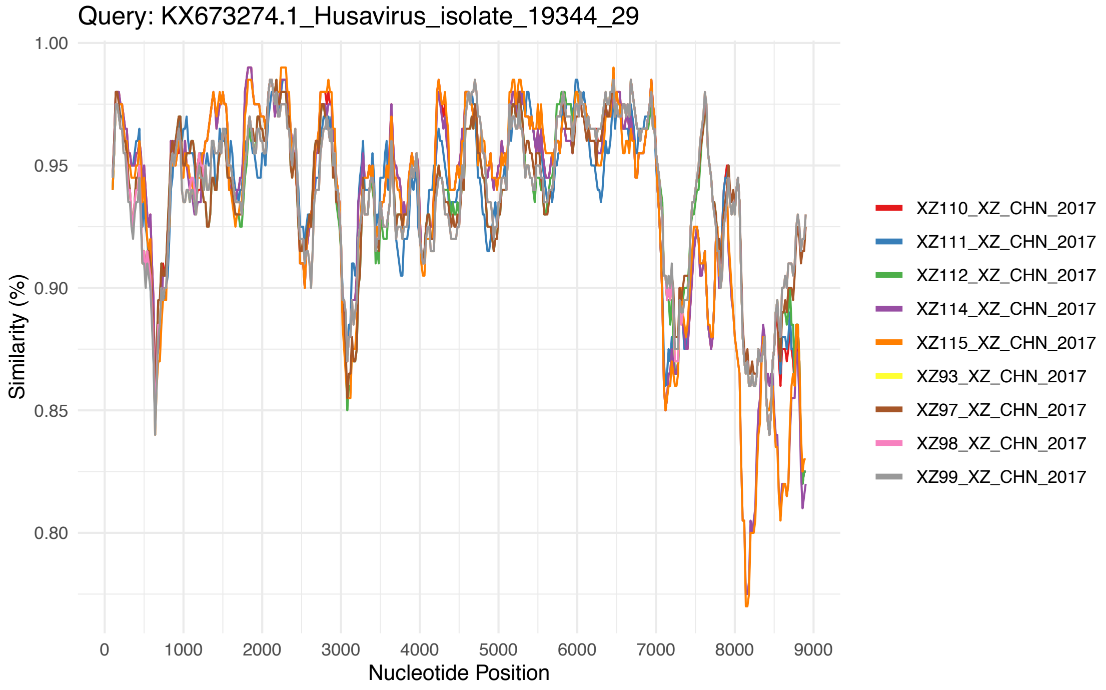

Figure S2. Nucleotide variation across the genome of nine husavirus strains (XZ93_XZ_CHN_2017, XZ97_XZ_CHN_2017, XZ98_XZ_CHN_2017, XZ99_XZ_CHN_2017, XZ110_XZ_CHN_2017, XZ111_XZ_CHN_2017, XZ112_XZ_CHN_2017, XZ114_XZ_CHN_2017 and XZ115_XZ_CHN_2017) relative to the reference strain (KX673274.1_Husavirus_isolate_19344_29). (A) XZ110_XZ_CHN_2017 vs. KX673274.1_Husavirus_isolate_19344_29; (B) XZ111_XZ_CHN_2017 *vs.* KX673274.1_Husavirus_isolate_19344_29; (C) XZ112_XZ_CHN_2017 *vs.* KX673274.1_Husavirus_isolate_19344_29; (D) XZ114_XZ_CHN_2017 *vs.* KX673274.1_Husavirus_isolate_19344_29; (E)

XZ115_XZ_CHN_2017 *vs.* KX673274.1_Husavirus_isolate_19344_29; (F)

XZ93_XZ_CHN_2017 *vs.* KX673274.1_Husavirus_isolate_19344_29; (G)

XZ97_XZ_CHN_2017 *vs.* KX673274.1_Husavirus_isolate_19344_29; (H)

XZ98_XZ_CHN_2017 *vs.* KX673274.1_Husavirus_isolate_19344_29; (I)

XZ99_XZ_CHN_2017 *vs.* KX673274.1_Husavirus_isolate_19344_29.

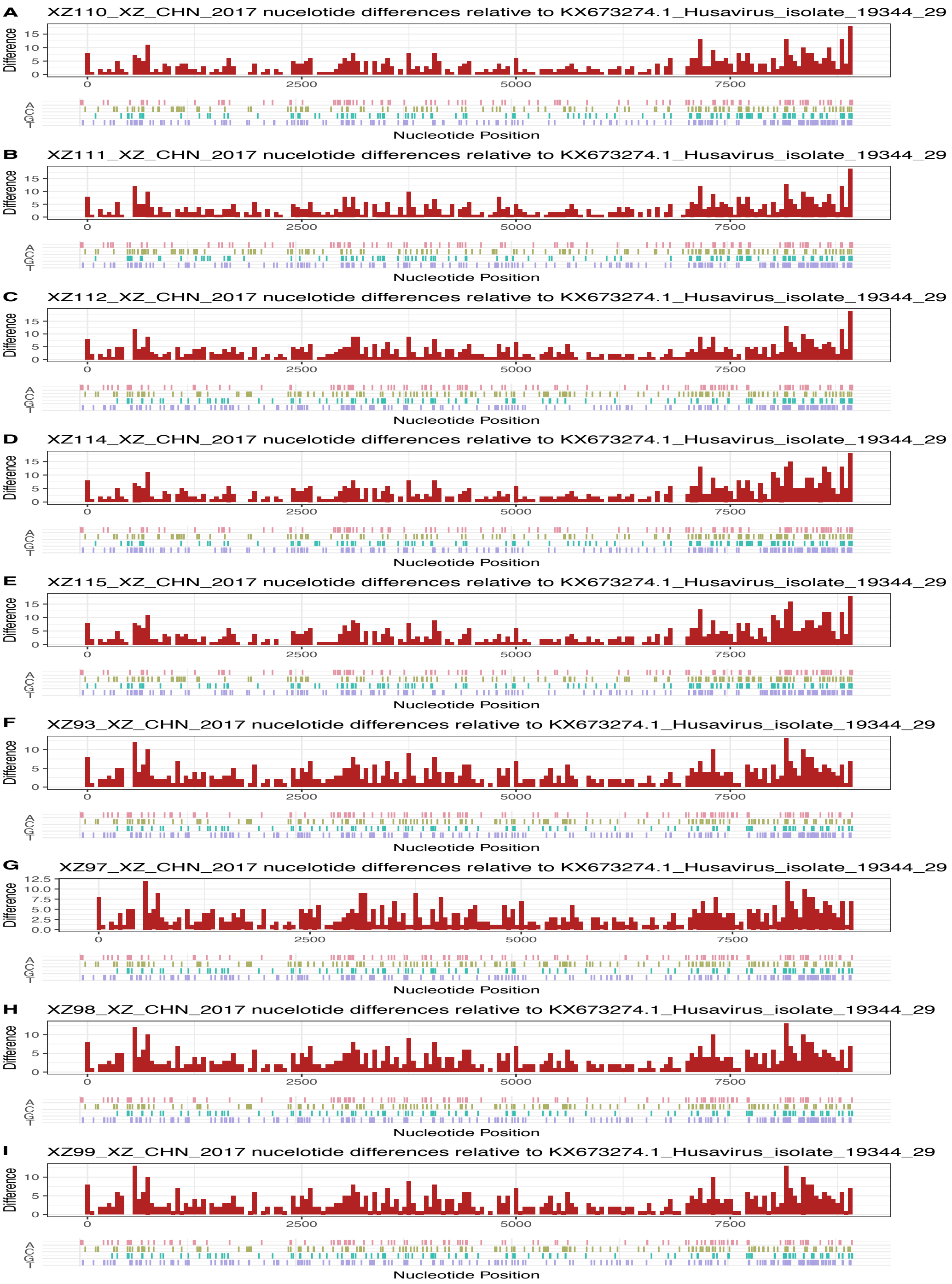

Table S1. The primers used for amplification and sequencing

Primer	Nucleotide position (nt)	Primer sequence (5’-3’)	Orientation
5’RACE-inner-primer	550	AGG TGA TGC TCC TCC ACC CAC TTC	Reverse
5’RACE-outer-primer	650	AAT TAC TCA CTA CCG CTA CAA TCG	Reverse
husa-60F	60	CCT CCT CTG GCA GTT CTC TGG ACA	Forward
husa-1060R	1060	CCA GCG CAC TCG TGA TCG CCC ACG	Reverse
husa-910F	910	GGA AAA CGC GTC CTG ATA GGG AGC	Forward
husa-1920R	1920	GGG ATC ATC ACA TAC ACT CAA TGC	Reverse
husa-1780F	1780	GAA GAG CGT CGA CAC TGT GGT GTC	Forward
husa-2780R	2780	CGC TTA GAT ACG AGC TTC CAA TCG	Reverse
husa-2630F	2630	GAG TTG CTG ATT AAG TCC GAC GAT	Forward
husa-3670R	3670	TGG CCA TCC TTG ATG AAT ATA TGG	Reverse
husa-3520F	3520	GAA CTT GAG CAG CTG TAT GGC TCT	Forward
husa-4580R	4580	ACA TGC TGG TGA TGT TTG GAC GCG	Reverse

husa-4380F	4380	CTG GAG GTG AAT GGG GTC CCT GCA	Forward
husa-5420R	5420	CGA AAT CGT ACT TAC CGC GGA GTC	Reverse
husa-5300F	5300	CGT TCA AGT GGC GTG AAC TGA CTG	Forward
husa-6300R	6300	GTA GGG TCT TCT CTC TCA ACA GAC	Reverse
husa-6140F	6140	GTG ATG ATG TGC CTG AAT TCA ATG	Forward
husa-7150R	7150	CTG GTA CGG CAC CTT CAA CTT CGC	Reverse
husa-6980F	6980	CAG GTA CAT GAC CTA CTG GCG TGG	Forward
husa-7990R	7990	GGA GAA ACT CAG TCG CGC AAT TCC	Reverse
husa-7760F	7760	GGA GTG TCA CCT AGT GAT ACA GCC	Forward
husa-8790R	8790	TAA ACG AAC TCG CTC TGG TAC GGC	Reverse
husa-8580F	8580	CAA TTC GGT ACA GGG TGT TCT GTC	Reverse
7500A		GGGGACCACTTTGTACAAGAAAGCTGGG$(T)_{24}$	Reverse

Table S2. The genomic sequence identity percentage of nucleotide and amino acid sequences, including the nine strains in this study and a reference strain 19344_29 (GenBank accession number KX673274). The left bottom of the table shows the nucleotide identity between each sequence, and the right top of the table presents the amino acid identity percentage.

	KX673274.1_Husavirus_isolate_19344_29	XZ110_XZ_CHN_2017	XZ111_XZ_CHN_2017	XZ112_XZ_CHN_2017	XZ114_XZ_CHN_2017	XZ115_XZ_CHN_2017	XZ93_XZ_CHN_2017	XZ97_XZ_CHN_2017	XZ98_XZ_CHN_2017	XZ99_XZ_CHN_2017
KX673274.1_Husavirus_isolate_19344_29	NA	0.969	0.97	0.972	0.966	0.965	0.974	0.975	0.974	0.973
XZ110_XZ_CHN_2017	0.938	NA	0.985	0.98	0.992	0.987	0.975	0.977	0.975	0.974
XZ111_XZ_CHN_2017	0.934	0.97	NA	0.982	0.979	0.978	0.977	0.978	0.977	0.977
XZ112_XZ_CHN_2017	0.935	0.958	0.961	NA	0.974	0.974	0.991	0.993	0.991	0.991
XZ114_XZ_CHN_2017	0.933	0.988	0.959	0.947	NA	0.994	0.969	0.971	0.969	0.968
XZ115_XZ_CHN_2017	0.932	0.981	0.959	0.947	0.991	NA	0.969	0.97	0.969	0.968

XZ93_XZ_CHN_2017	0.937	0.954	0.956	0.987	0.943	0.942	NA	0.994	1	0.999
XZ97_XZ_CHN_2017	0.937	0.953	0.955	0.991	0.942	0.941	0.992	NA	0.994	0.993
XZ98_XZ_CHN_2017	0.937	0.954	0.956	0.987	0.943	0.942	0.999	0.993	NA	0.999
XZ99_XZ_CHN_2017	0.937	0.954	0.955	0.987	0.943	0.942	0.999	0.992	0.999	NA
